# Supplementary material for: Live Cell Imaging of ATP Levels Reveals Metabolic Compartmentalization within Motoneurons and Early Metabolic Changes in FUS ALS Motoneurons
Source: Cells. 2023 May 9;12(10):1352. doi: 10.3390/cells12101352 (PMC10216752; doi:10.3390/cells12101352)
Supplement: Supplementary file 1 [file cells-12-01352-s001.zip › cells-2332525-supplementary.pdf]

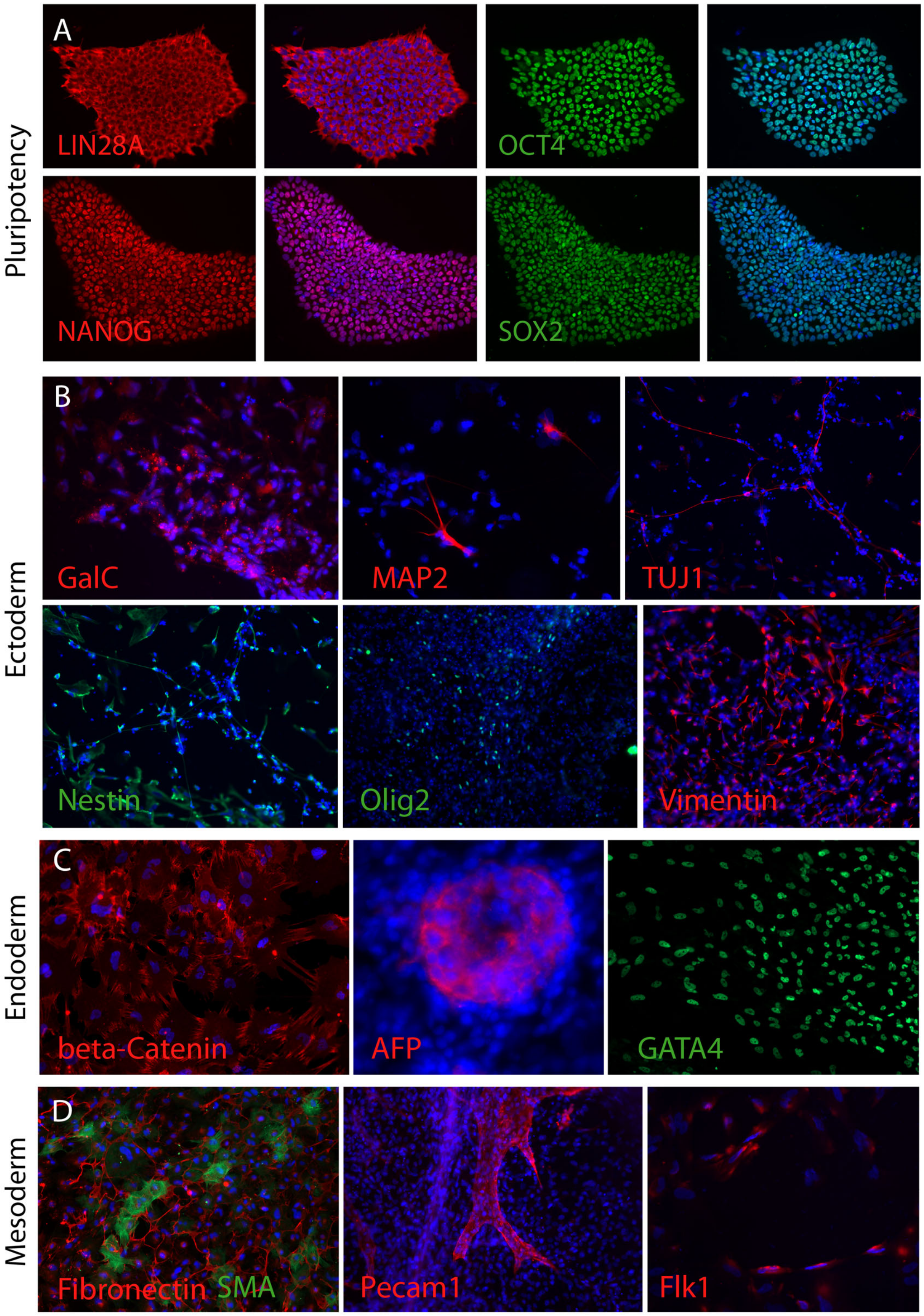

Figure S1. Initial characterization of the FUS-Q23L hiPSC line after fibroblast reprogramming. **(A)** hiPCs derived from patient fibroblasts were tested for presence of pluripotency markers by immunostainings for Nanog, Oct4, Sox2 and Lin24A (**B–D)** FUS-Q23L hiPSC colonies were able to differentiate into all three germ layers as shown by immunostainings for typical markers for Ectoderm **(B),** Endoderm **(C)** and Mesoderm **(D).** n ≥ 3 biological replicates**.**

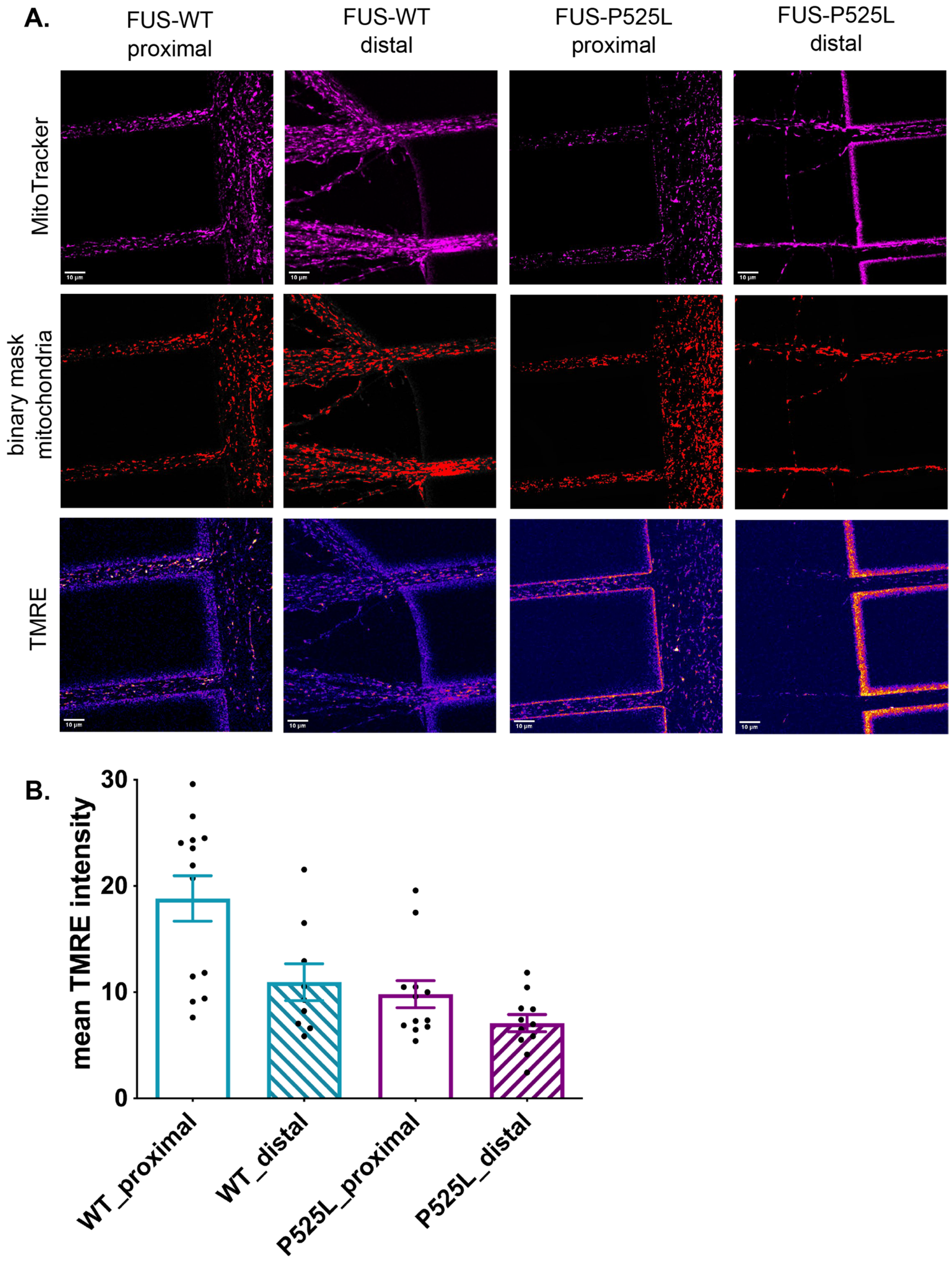

Figure S2. Measurement of mitochondrial membrane potential in proximal and distal axons using TMRE**.** **(A)** Motor neurons were seeded into MFCs and axons were allowed to grow through the micro channels for two weeks of maturation. Then, neurons were stained with MitoTracker deep red and TMRE for subsequent live cell imaging. Upper panel: MitoTracker staining. Middle panel: binary mask of mitochondria created in Fiji. lower panel: TMRE staining. Scale bar indicates 10 µm. (**B)** Quantification of mean TMRE signal intensity within mitochondria. Data indicated as mean ± SEM (N=2).

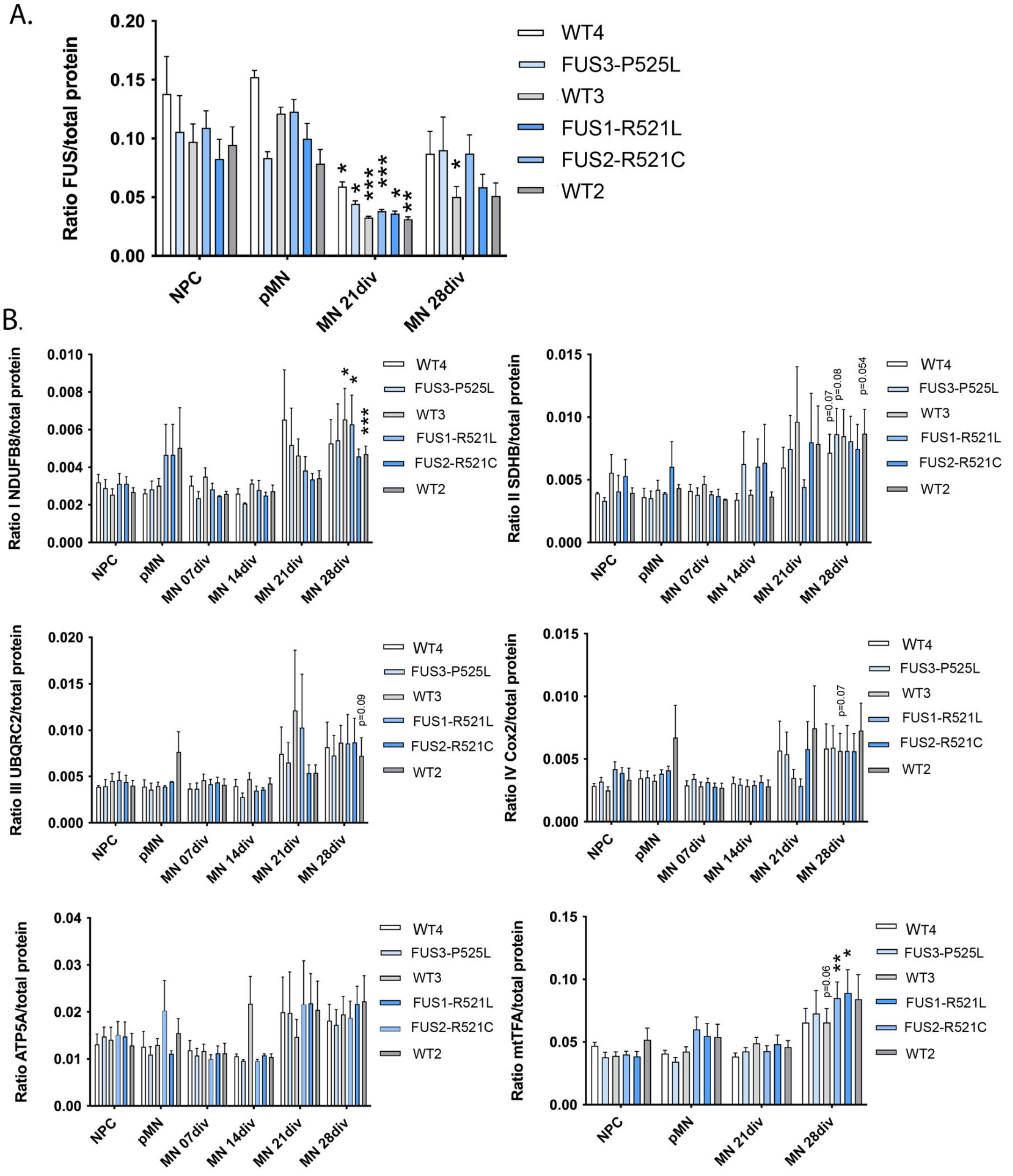

Figure S3. Western blot analysis of FUS and mitochondrial proteins during FUS-ALS MN differentiation and maturation. **(A)** Quantification of western blots for FUS protein levels during MN differentiation and maturation in FUS-ALS lines. n ≥ 4 biological replicates **(B)** Quantification of western blots for selected components of the mitochondrial respiratory chain subunits I-V: NDUFB8, SDHB, UQCRC2, COX2, ATP5A, as well as mitochondrial transcription factor A(mtTFA) show increase in the relative amounts of these proteins (excluding ATP5A) in 28 day old MNs in FUS-ALS lines. Asterisks show significance comparing values between different stages of differentiation within the same line (n ≥ 3 biological replicates).

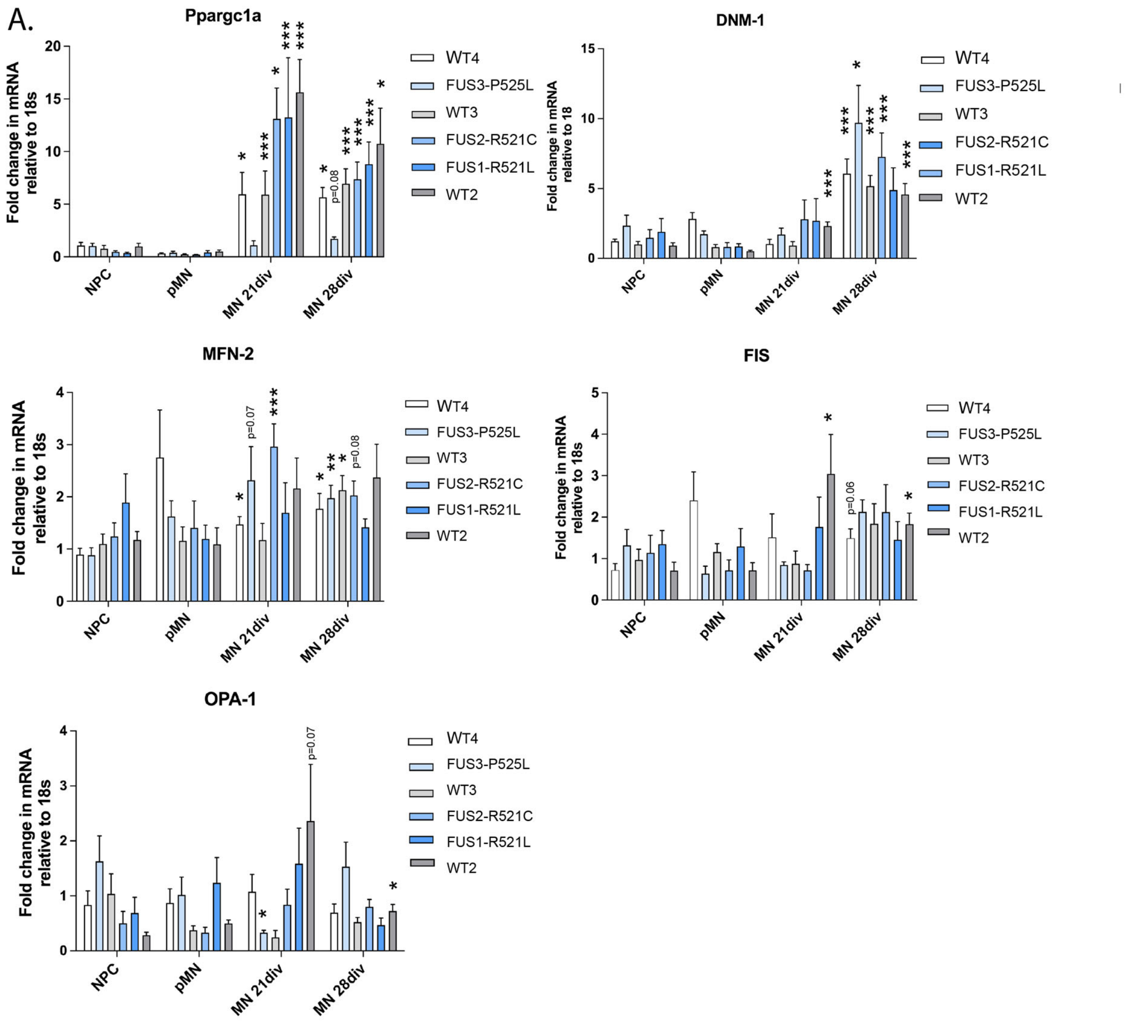

Figure S4. Changes in expression levels of mRNAs important for mitochondrial function during FUS-ALS MN differentiation and maturation**. (A)** qPCR quantification of relative expression levels in several mRNAs critical for maintenance and operation of the mitochondrial network: PPARGC-1α, Dnm1L, MFN-2, OPA1 and FIS1 during MN differentiation and maturation in FUS-ALS lines. Asterisks show significance comparing values between different stages of differentiation within the same line. (n ≥ 5 biological replicates, n ≥ 2 technical replicas).

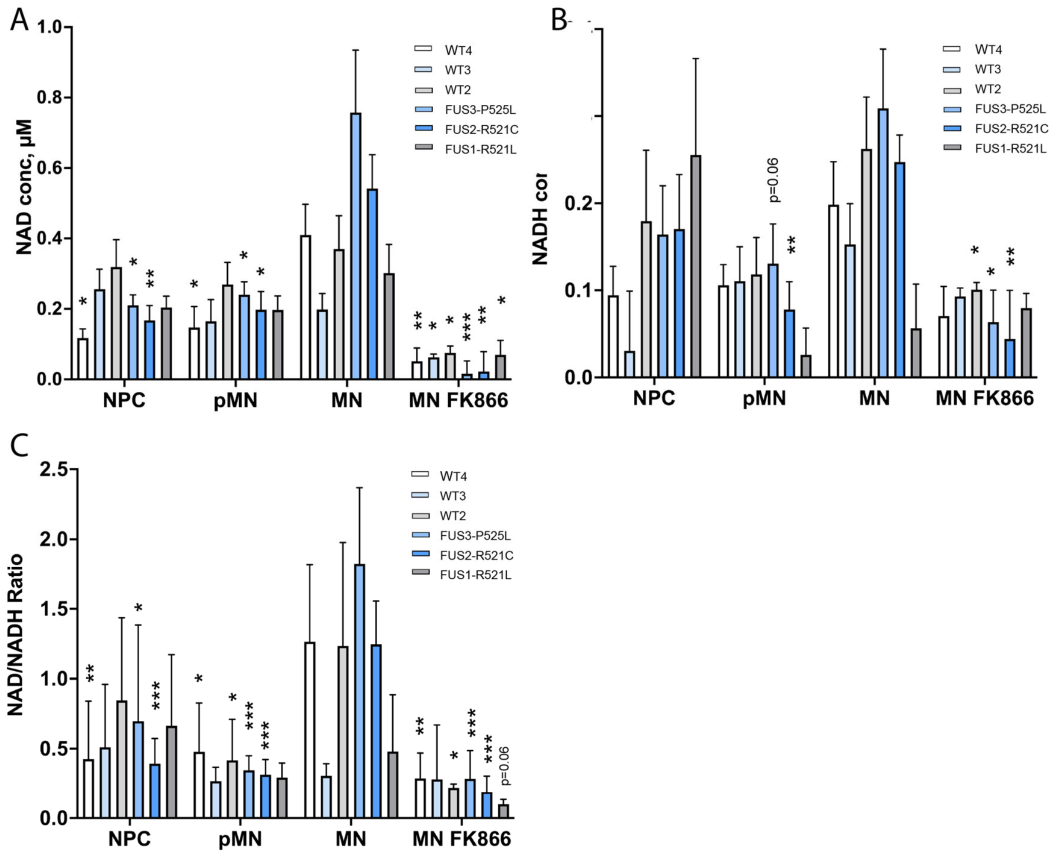

Figure S5. NAD+/NADH levels and redox ratio during MN differentiation of FUS-ALS cultured motoneurons. (**A–C**) Measurements of NAD+ **(A)**, NADH **(B)** concentrations and their redox ratios **(C)** across all tested individual control and FUS mutant lines at different stages of MN differentiation. Significances are shown as * between MN stage vs other stages or treatment within the same MN (n ≥ 5 biological replicates per line).

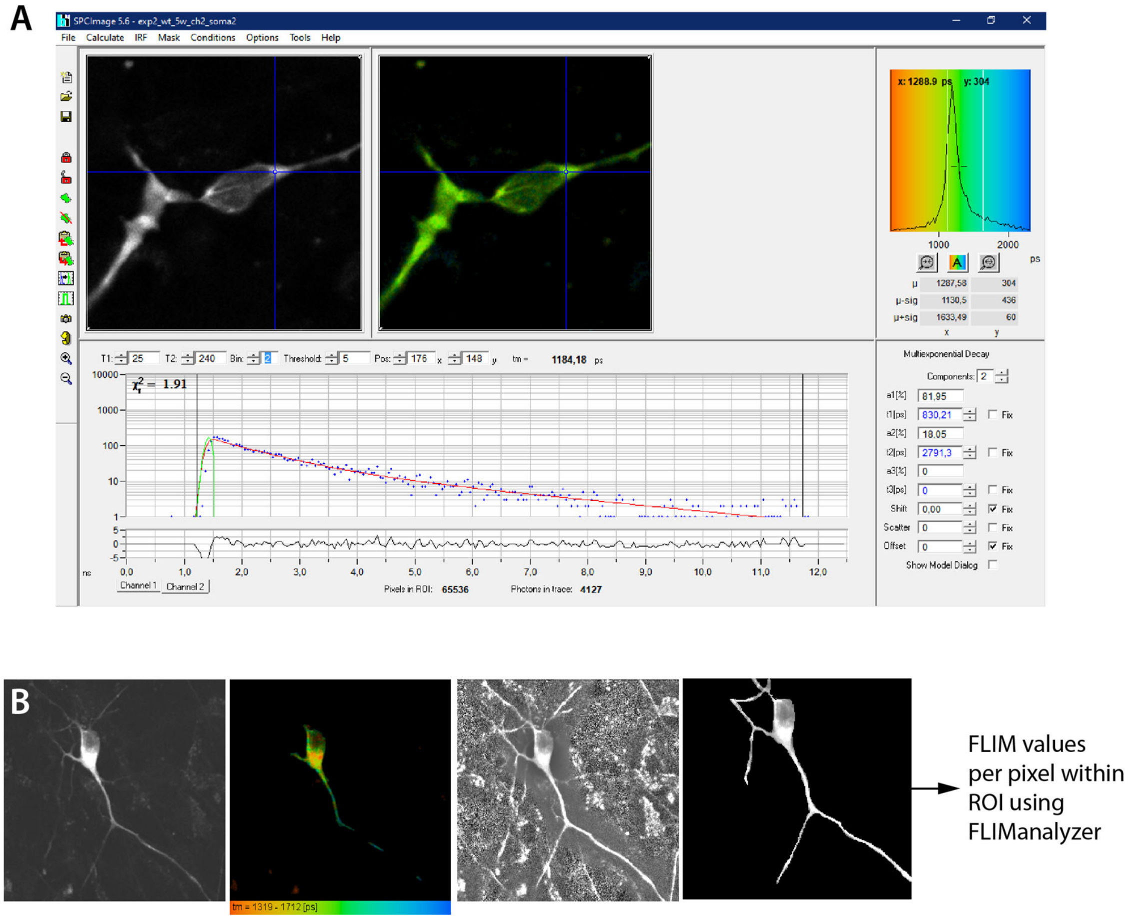

Figure S6. Workflow of FLIM values extraction from single pixel and individual compartments ROI. (**A**) Snapshot of SPCImage software window with an example curve fitting for single pixel FLIM measurements. Modelling is based on two components incomplete model fitting. (**B**) Fluorescence donor (CFP) intensity signal, color coded FLIM values per pixel, and contrast-guided selection of ROI for data extraction to exclude nuclei and background noise measurements.

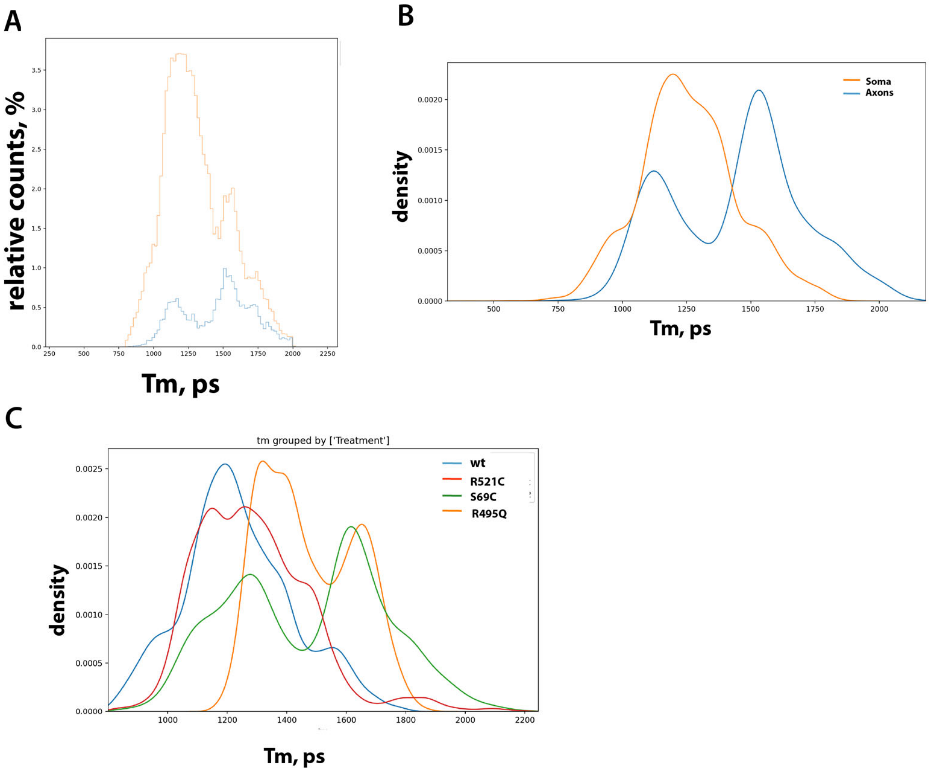

Figure S7. Comparison of individual pixel Tm values distributions in FLIM measurements between somatic and axonal compartments. (**A**) Frequency distribution histogram (**A**) and normalized KDE distribution plot (**B–C**) comparing Tm values of somatic and axonal compartments (**B**), as well as comparing control (wt1) and mutant FUS MN.

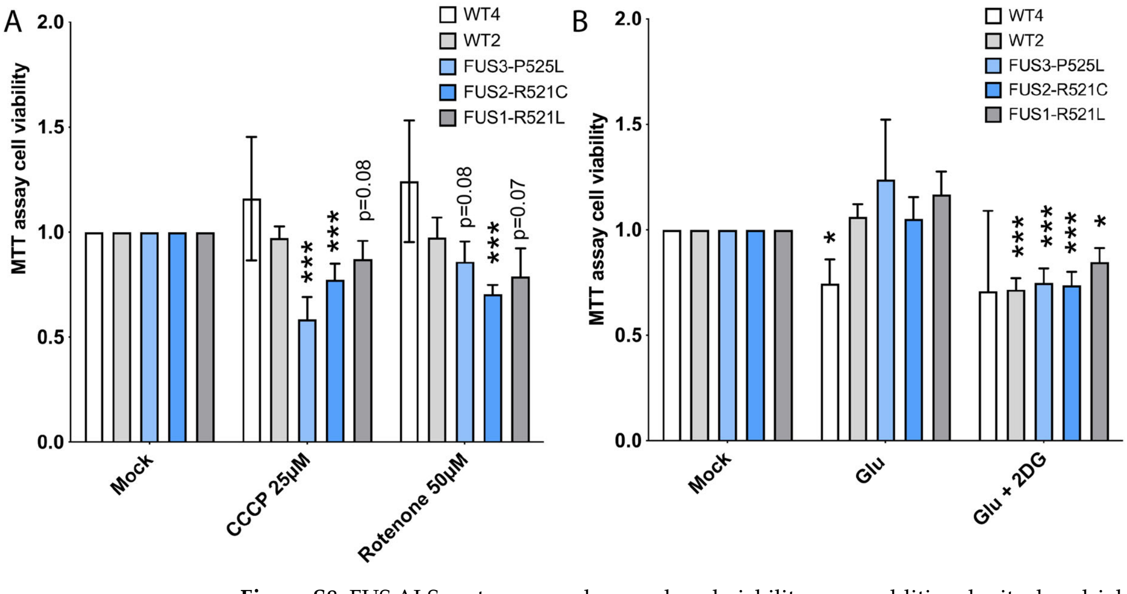

Figure S8. FUS ALS motoneurons have reduced viability upon additional mitochondrial stress. **(A)** Bar graphs comparing cell survival of individual control and FUS ALS mutant MNs using MTT cell viability assay treated with mitochondrial inhibitors CCCP and Rotenone. (**B)** Bar graphs comparing cell survival of individual control and FUS ALS mutant MNs using MTT cell viability assay after glutamate stimulation or blocking glycolysis with 2-deoxy-d-glucose (2DG) (n ≥ 3 biological replicates per line).
